# Insights into the keratin efficient degradation mechanism mediated by *Bacillus* sp. CN2 based on integrating functional degradomics

**DOI:** 10.1186/s13068-023-02308-0

**Published:** 2023-04-04

**Authors:** Yuhong Lai, Xiuyun Wu, Xianliang zheng, Weiguang Li, Lushan Wang

**Affiliations:** 1 State Key Laboratory of Microbial Technology, Institute of Microbial Technology, Shandong University, 72 Binhai Road, Qingdao, 266237 Shandong China; 2 Angel Yeast Inc, Yichang, 443003 Hubei China

**Keywords:** *Bacillus* sp. CN2, Keratinous waste, Microbial protease, Keratinolysis, T3 γ-glutamyltransferase

## Abstract

**Background:**

Keratin, the main component of chicken feather, is the third most abundant material after cellulose and chitin. Keratin can be converted into high-value compounds and is considered a potential high-quality protein supplement; However, its recalcitrance makes its breakdown a challenge, and the mechanisms of action of keratinolytic proteases-mediated keratinous substrates degradation are not yet fully elucidated. *Bacillus* sp. CN2, having many protease-coding genes, is a dominant species in keratin-rich materials environments. To explore the degradation patterns of feather keratin, in this study, we investigated the characteristics of feather degradation by strain CN2 based on the functional-degradomics technology.

**Results:**

*Bacillus* sp. CN2 showed strong feather keratin degradation activities, which could degrade native feathers efficiently resulting in 86.70% weight loss in 24 h, along with the production of 195.05 ± 6.65 U/mL keratinases at 48 h, and the release of 0.40 mg/mL soluble proteins at 60 h. The extracellular protease consortium had wide substrate specificity and exhibited excellent biodegradability toward soluble and insoluble proteins. Importantly, analysis of the extracellular proteome revealed the presence of a highly-efficient keratin degradation system. Firstly, T3 γ-glutamyltransferase provides a reductive force to break the dense disulfide bond structure of keratin. Then S8B serine endopeptidases first hydrolyze keratin to expose more cleavage sites. Finally, keratin is degraded into small peptides under the synergistic action of proteases such as M4, S8C, and S8A. Consistent with this, high-performance liquid chromatography (HPLC) and amino acid analysis showed that the feather keratin hydrolysate contained a large number of soluble peptides and essential amino acids.

**Conclusions:**

The specific expression of γ-glutamyltransferase and co-secretion of endopeptidase and exopeptidase by the *Bacillus* sp. CN2 play an important role in feather keratin degradation. This insight increases our understanding of the keratinous substrate degradation and may inspire the design of the optimal enzyme cocktails for more efficient exploration of protein resources in industrial applications.

**Supplementary Information:**

The online version contains supplementary material available at 10.1186/s13068-023-02308-0.

## Background

According to statistics, approximately 10 million tons of keratin waste are produced each year worldwide, of which feather waste constitutes approximately 8.5 million tons. Feathers contain more than 85% crude protein and 70% amino acids, as well as some high-value elements, vitamins and growth factors [1]. Recently, researchers have shown interest in applying these biomaterials to the development of various products (such as feed [2], fertilizer [3], and biofilm [4]). As members of the intermediate filament family of proteins, keratin is a type of fibrous protein with a high sulfur content, and constitutes the third most abundant refractory polymer in nature following cellulose and chitin [5–7]. As a result of the abundant hydrophobic amino acid residues on the surface of keratin, and the existence of hydrophobic interactions, hydrogen bonds, and intra-/intermolecular disulfide cross-linking, keratin exhibits high mechanical stability and is not easily degraded [8]. Energy-intensive hydrothermal processes are currently used to manage feathers. This strategy is not only costly, but also results in the loss of thermolabile amino acids and yields the lysino-alanine and lanthionine, which reduces digestibility and are potentially toxic [9–11].

In recent years, bioconversion of keratin waste into valuable products has become a “green” concept in sustainable industry [12, 13]. The emergence of keratinolytic microorganisms and keratinases has made it possible to efficiently transform keratin. Microbial keratinase producers are ubiquitously found in nature, including bacteria, actinomycetes, and fungi [14]. Bacteria are major players in keratin degradation and have been the most intensively studied to date. Among them, *Bacillus* species*,* such as *Bacillus licheniformis*, *Bacillus subtilis*, *Bacillus amyloliquefaciens*, are capable of producing keratinase [15–17]. The advantages of using keratinases to degrade keratinous wastes are low-cost, environmentally friendly, and high quality by-products compared to the hydrothermal treatment [18, 19]. For instance, the supplementation of animal feed with keratinase was reported to improve nutrient digestibility, palatability, and immune response [20, 21]. Keratin hydrolysate obtained from keratin hydrolysis by keratinase is rich in ammonia and amino acids and hence, can be exploited as biofertilizer [19]. However, the mechanism of efficient degradation of keratin by these microorganisms has not been fully elucidated, which greatly hinders the sustainable utilization and industrial development of keratin waste.

The ability to secrete a variety of keratinases (EC 3.4.21/24/99.11) is the common characteristic of keratinolytic microorganisms [20, 22]. However, the degradation of keratin driven by microbes is a complex process, in which keratinases play an important, but not exclusive, role [16]. Based on an understanding of the structure of keratin, it is generally considered that the microbial decomposition of keratin can be divided into three main stages: denaturation, degradation and transamination [17]. At present, the main challenge in the application of keratinase is not only low activity following the separation or heterologous expression of the enzyme derived from microorganisms, but also the impenetrable nature of the substrate, which makes it difficult to effectively hydrolyze [23]. Although the hydrolytic activity of keratinase on keratin monomers is higher than that of ordinary proteases, complete degradation of keratin requires the synergistic action of multiple enzymes and a single keratinase appears to have difficulty efficiently hydrolyzing natural keratin substrates with a complex structure [24, 25]. Systematic research using functional omics technology indicates that the degradation of keratin is an efficient and orderly coordinated catalytic process involving the participation of multiple enzymes dominated by extracellular keratinase.

The degradome defines the complete repertoire of proteolytic enzymes found in a cell, tissue or organism at any particular moment or circumstance [26]. The growing amount of protease data available in public databases and the determination of an increasing volume of genomic data has accelerated degradome research, and made it possible to predict the degradome of a species at the genomic or proteomic level [27]. Importantly, the functional keratinase in the degradome that plays a role in the degradation of keratin can be accurately identified using proteomic methods, which provides theoretical guidance for the large-scale industrial biodegradation of keratin [28, 29].

We previously identified a proteolytic strain, *Bacillus* sp. CN2, which exhibited a potent protein degradation system [30]. In this study, the keratinolytic potential of strain CN2 was evaluated using feather waste as the only nitrogen source. The functional enzyme consortium, substrate specificity, degradation products of keratin, and mode of degradation of feather keratin by this strain were investigated. Our data indicate that the keratinase activity of strain CN2 is significantly higher than those in most other microorganisms analyzed, such as *B. licheniformis* ALW1 [31], *B. cereus* B5esz [32], and *B. subtilis* MBF11 [33]. Therefore, it is a promising candidate as a producer of effective keratinases in technologies for keratinolytic processing of keratinous waste.

## Results

### Characterization of the feather keratin degradation system of *Bacillus* sp. CN2

**Fig. 1:**
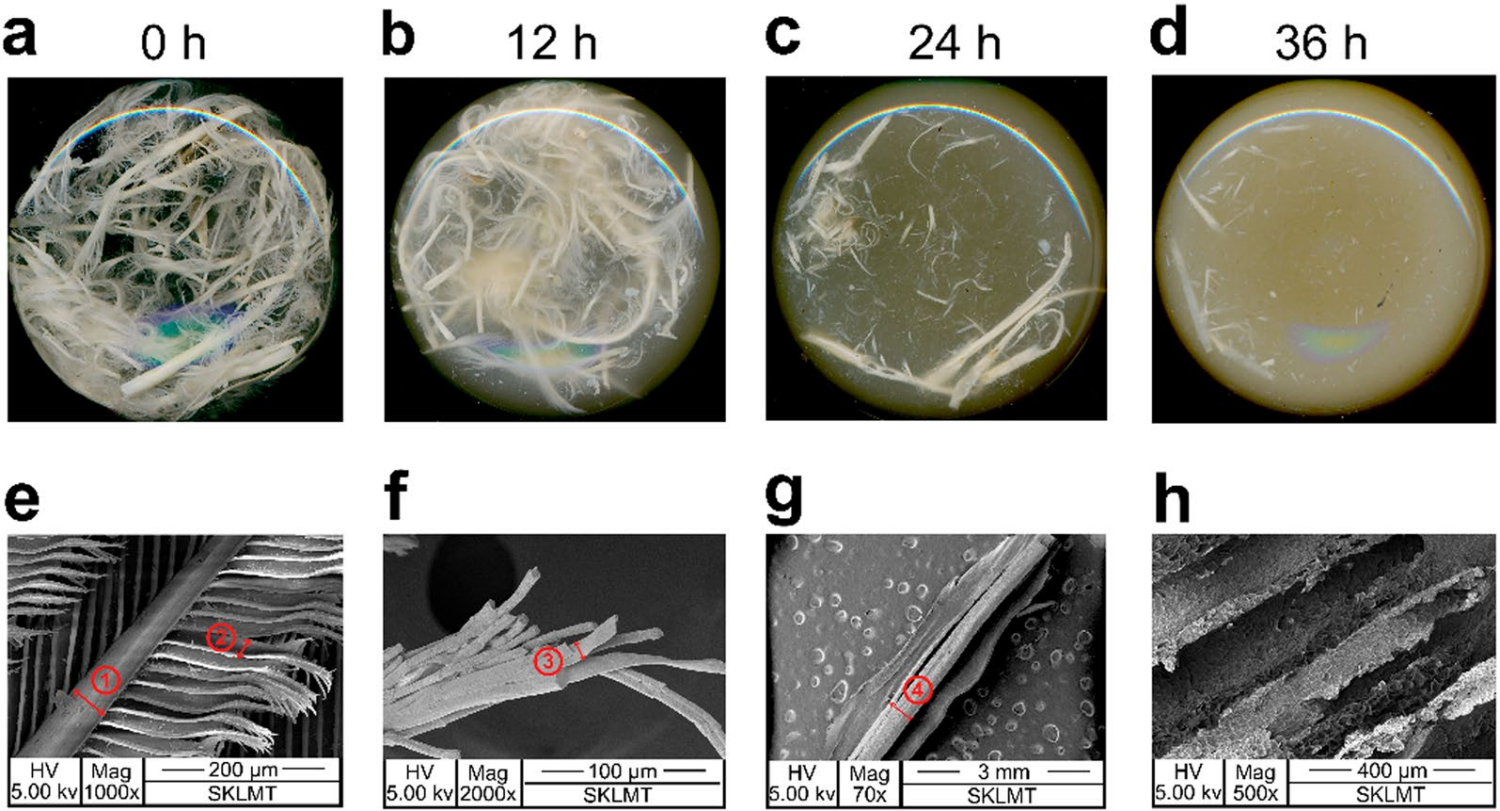
Degradation dynamics of feather keratin by *Bacillus* sp. CN2. **a**–**d** Biodegradation of intact chicken feathers by the keratinolytic bacterium CN2. **e**–**h** SEM to examine changes in feather morphology, where (**e**) is the intact feather, and **f**–**h** show the feathers after 12, 24, and 36 h of incubation, respectively. The red arrows indicate the change in diameter of the feather morphology

*Bacillus* sp. CN2 can degrade a variety of protein substrate, including wheat bran, corn bran, corn steep liquor, and maize protein powder [30]. In this study, a system was designed to degrade 10 g/L of feathers. The intact feather (pinna rachis 54 μm, 1000 × ; barbule 36 μm, 1000 × , Fig. 1e) showed signs of degradation during the early stage (12 h) of the fermentation (Fig. 1a and b). Degradation appeared as breakage of the barbule, exposure of the internal fiber structure (10 μm, 2000 ×), and gradual turbidity of the medium (Fig. 1f). By 24 h, the pinna rachis and branches were almost completely degraded, with only a few of the largest barbs (0.3 mm, 70 ×) remaining (Fig. 1c and g). By 36 h, the residual feathers were further degraded, the fermentation broth turned yellow–brown, and the rachis structure was completely destroyed (Fig. 1d and h). Beyond 48 h, complete degradation was achieved, and there were no observable structures remaining. The imaging of chicken feather degradation by *Bacillus* sp. FPF-1 in a previous report showed a similar pattern [34].

**Fig. 2:**
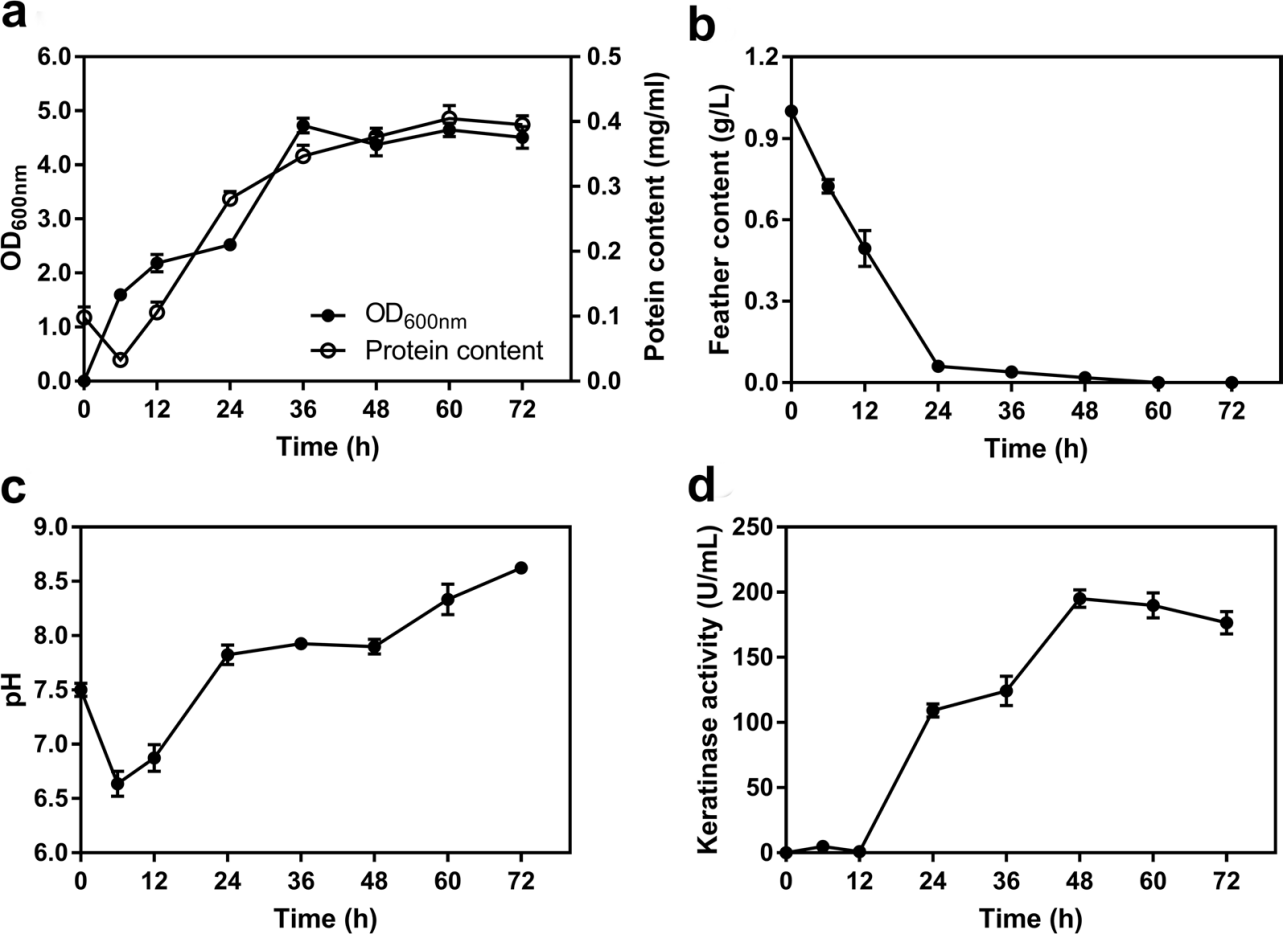
Time-courses of *Bacillus* sp. CN2 growth on feather keratins and degradation product accumulation. **a** Changes in cell density and soluble protein concentration of the degradation system. **b** Feather degradation rate. **c** Changes in pH of the degradation system. **d** Keratinase activity in the process of hydrolysis

The process of degradation of feather keratin and the physicochemical properties of the degradation system were monitored throughout the fermentation. The results showed that the decreased protein contents of the media supported rapid cell proliferation during the early stage (0–12 h) of the fermentation (Fig. 2a). The feathers were rapidly degraded within 24 h, and the degradation rate reached 86.70% (Fig. 2b). Along with a pungent ammonia smell, the pH of the medium increased from 7.43 at the start of fermentation to 8.51 after 72 h of incubation (Fig. 2c), which may reflect the increase in metabolites secreted by microorganisms and the free NH_4_^+^ produced by proteolysis. Intriguingly, keratinase activity was low at the beginning (12 h) of the fermentation (Fig. 2d), significantly increased by 24 h, then peaked, along with the enzyme concentration, at 48 h with keratinase activity of 195.05 ± 6.65 U/mL. Beyond 48 h of incubation, the keratinase yield decreased slightly, and 176.48 ± 8.64 U/mL of keratinase activity was detected at 72 h of incubation. The produced protease acts on feather keratin, resulting in the degradation of keratin and the production of a large amount of soluble protein (~ 0.40 mg/mL).

### Dynamic enzyme profile and analysis of the degradation potential of extracellular protease

**Fig. 3:**
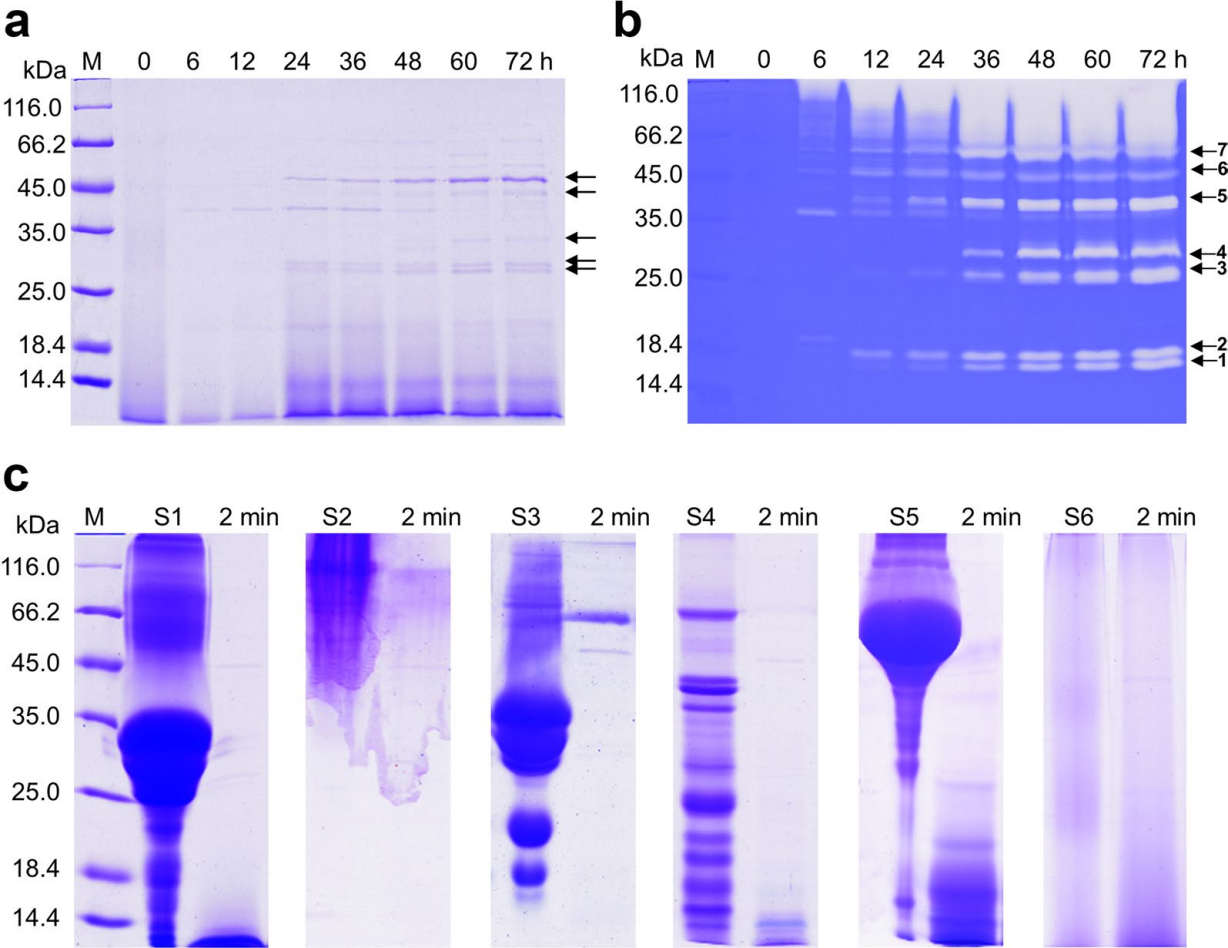
Analysis of the extracellular proteins, proteases, and the hydrolysis effects of different natural substrates. **a** Dynamic changes of extracellular proteins. **b** Sequential changes of extracellular proteases. **c** Hydrolysis potential of extracellular functional enzymes to different natural substrates. The seven arrowheads indicate the bands of major proteases. M, protein molecular weight markers; S, substrate, S1–S6 represent 2% casein, gelatin, nonfat-dried milk, soybean cake meal, BSA, and feather powder, respectively. Feather powder was ground from intact chicken feathers. Lane 2 indicates the interaction of the enzyme with different natural substrates for 2 min

Based on the significant feather keratin degradation ability of *Bacillus* sp. CN2, the secretion of extracellular protein and protease from this bacterium was detected. Strain CN2 secreted a variety of proteins when grown on the medium with feather keratin as the only nitrogen source, with one predominant protein having a molecular weight (MW) of 45 kDa and two other proteins of 30 kDa (Fig. 3a).

The secretion of most microbial proteases is regulated by substrate type, and keratinase degrades keratin into nutrients only under nutritional stress [21, 35]. Our results showed that extracellular protease secretion started at 6 h and the variety of proteases secreted increased continuously within 24 h when strain CN2 was growth on feathers as the substrate (Fig. 3b). After 36 h, the variety of extracellular protease species stabilized, mainly comprising seven protease bands, and the abundance of individual proteases increased slightly with fermentation time, which was similar to the results of a previous study [30]. Based on our observation of almost complete degradation of feathers within 24 h, it can be speculated that the type of protease, rather than the abundance, plays a key role in the efficient degradation of keratin. This mode was also found in the degradation of feather and bristle by *Onygena corvina* [36].

To explore the substrate degradation potential of the *Bacillus* sp. CN2 extracellular functional enzyme consortium, the hydrolysis of six different protein substrates was detected. As shown in Fig. 3c, the crude enzyme extracted from the 72 h fermentation broth of strain CN2 exhibited the strongest hydrolytic ability against casein (20 < MW < 38 kDa), gelatin (MW > 40 kDa), nonfat-dried milk (25 < MW < 35 kDa), and soybean cake powder (10 < MW < 60 kDa) within 2 min, followed by BSA (40 < MW < 66 kDa), and feather powder. Larger amounts of endopeptidase, which may exist in the extracellular functional enzyme consortium, exhibited a greater hydrolytic effect on the soluble proteins, resulting in no significant residual protein bands being detected in the hydrolysate. However, large molecules of BSA were quickly hydrolyzed to smaller polypeptides (< 18 kDa) within 2 min, and the further degradation of small peptides may take longer. It is speculated that the difference in degradation rate may be caused by the substrate type. However, the protease in the culture media did not show any activity towards native feather powder (Fig. 3c), which may be because the keratinase, when detached from living cells, lacks catalytic activity against feather keratins [23]. In conclusion, CN2 extracellular enzymes have wide substrate specificity, but the hydrolysis of feather keratin requires the presence of living cells or reducing agents and other cofactors.

### Characteristics of feather degradation by extracellular enzymes

**Fig. 4:**
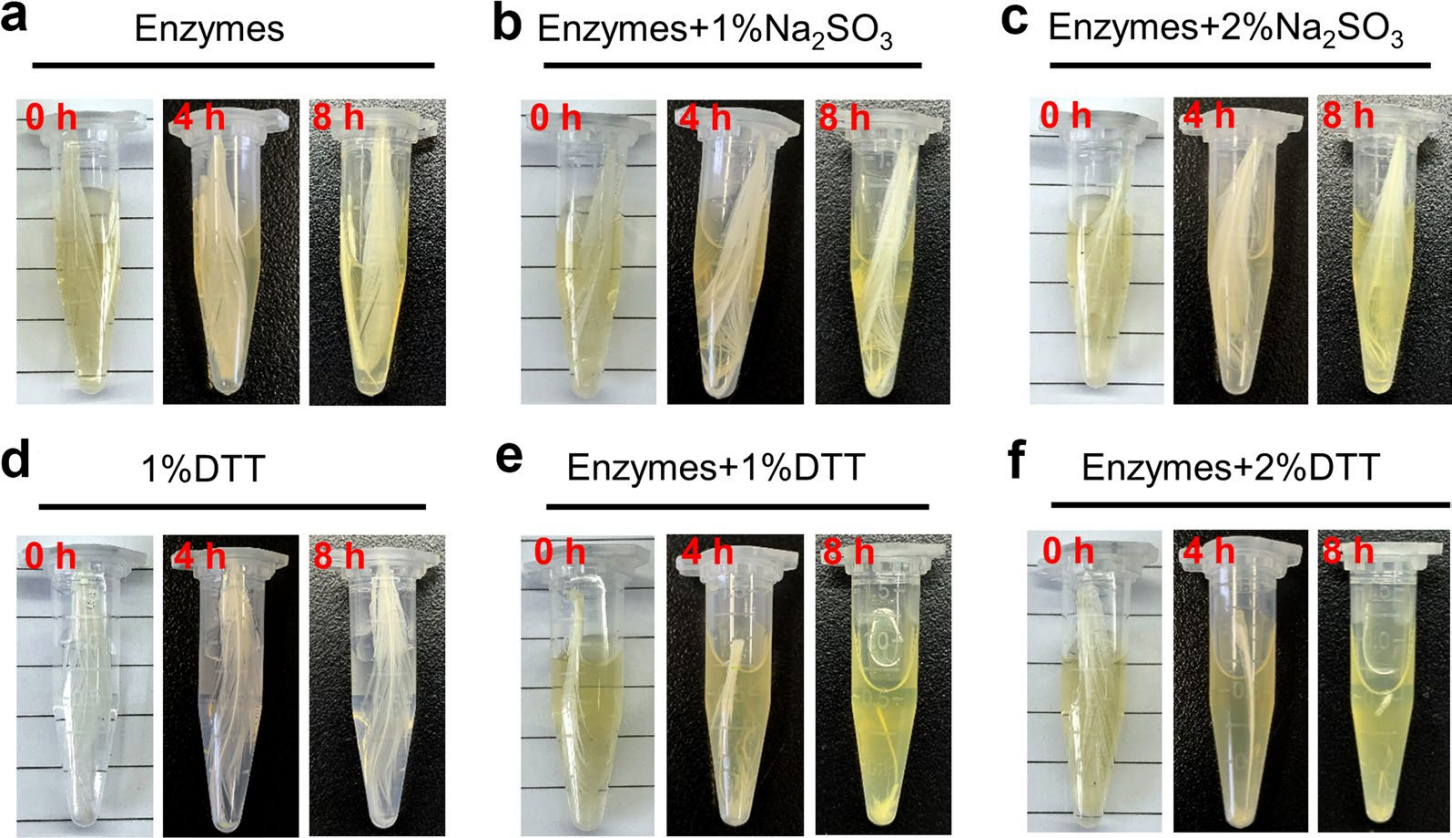
In vitro detection of the effect of reducing agents on the feather hydrolysis by keratinase. Na_2_SO_3_ and DTT were used as reducing agents. Cell-free fermentation broth at 72 h was collected and used as a crude enzyme for hydrolyzing feather keratin

Feather keratin has a dense structure that acts as a hydrophobicity resistance barrier, and the breaking of disulfide bonds is the rate-limiting step for its degradation [24]. It has been reported that the degradation of keratins in some *Bacillus* species relies on extracellular proteases and sulphitolytic systems [17, 37]. To verify whether sulfite is the reducing force for strain CN2 to break disulfide bonds, the effect of reducing agents on promoting the hydrolysis of feather keratin by keratinase in vitro was studied. The extracellular enzyme obtained was incubated with 1% (*w/v*) feathers for 8 h at 200 rpm and at 40 °C, and no morphological changes were observed in the feathers (Fig. 4a). In the control group, feathers were immersed in 1 mL of 1% or 2% sodium sulfite and DTT for the same reaction, with similar results (Fig. 4d), which was consistent with previous research [38]. When sulfite was used as a reducing agent, keratinase in the synergistic system could not degrade feather keratin (Fig. 4b and c). This result was different from the reported phenomenon that sulfites provide reducing power to break the cysteine disulfide bonds in feather keratin throughout the whole fermentation process [16]. However, when 1% DTT was added to the extracellular crude enzyme reaction for 4 h, the barbules were significantly degraded, and the feathers were almost completely degraded after 8 h (Fig. 4e), and 2% DTT enhanced this effect further (Fig. 4f).

The above phenomenon was further confirmed by electrochemical detection of the cysteine content in the synergistic system of crude enzyme and reducing agent (Additional file 1: Fig. S1). With increased reaction time for the fermentation broth, DTT, and feather keratin, the cysteine content in the system gradually increased, suggesting that the large number of disulfide bonds was destroyed during keratin degradation, and the reducing power provided by DTT plays an important role in the hydrolysis of feathers by keratinase.

### Changes to the sulfur compounds in the degradation system

**Fig. 5:**
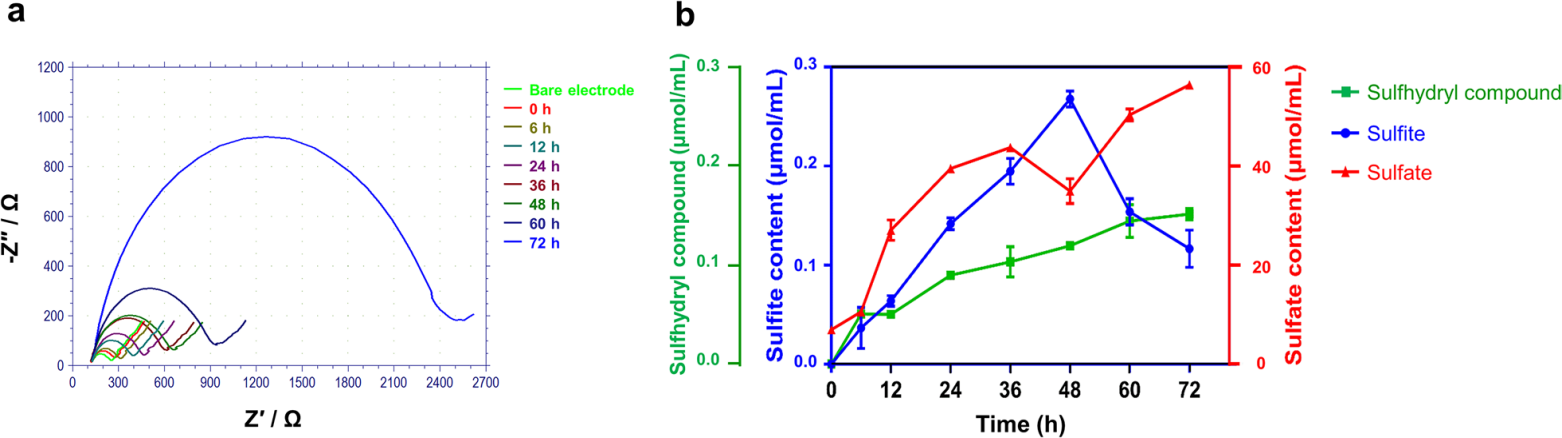
Changes in the content of sulfur compounds and cysteine in the degradation system. **a** The content of cysteine in the reaction system was detected by electrochemical detection. **b** Changes in the content of three sulfur compounds during the degradation of keratin

Opening the dense disulfide bond structure of keratin is crucial for feather degradation, and involves the production of cysteine. During the 0–72 h fermentation, the concentration changes in three sulfur compounds (sulfate, sulfite, and sulfhydryl compound) and cysteine were detected every 12 h, and the results are shown in Fig. 5. The concentration of sulfhydryl compounds remained at the lowest level throughout the experiment, but the content of cysteine increased continuously in the fermentation broth, as determined by electrochemical detection (Fig. 5a), suggesting that the disulfide bonds of keratin are continuously destroyed. High concentrations of cysteine are known to be toxic to cells, so cysteine is continuously absorbed by bacterial cells to synthesize the intermediate metabolite L-cysteine-sulfinate as the substrate for sulfite formation [17, 39]. Sulfate (6.32 μmol/mL) was detected in the sterilized medium (0 h), but at a lower concentration. Following inoculation with strain CN2, the sulfate concentration in the fermentation broth increased significantly, indicating that the strain produced a large amount of SO_4_^2−^ during feather keratin fermentation. Sulfite was not detected in the culture medium before fermentation, and its concentration was low during the whole fermentation process, peaking at only 0.27 μmol/mL at 48 h (Fig. 5b). A potential explanation for this phenomenon is that sulfites are rapidly oxidized to sulfate by some factor in the environment, rather than acting as a reducing force to break keratin disulfide bonds, which was also confirmed by in vitro degradation experiments of keratin.

In conclusion, although *Bacillus* sp. CN2 can continuously produce sulfate, the content of sulfite is low, it is indicated that another reducing agent may be operating to degrade the keratin structure and expose more keratinase or other protease cleavage sites, which is different from the observations of previous research [17].

### Transcriptomic and proteomic detection of keratin degradation-related enzyme components

**Fig. 6:**
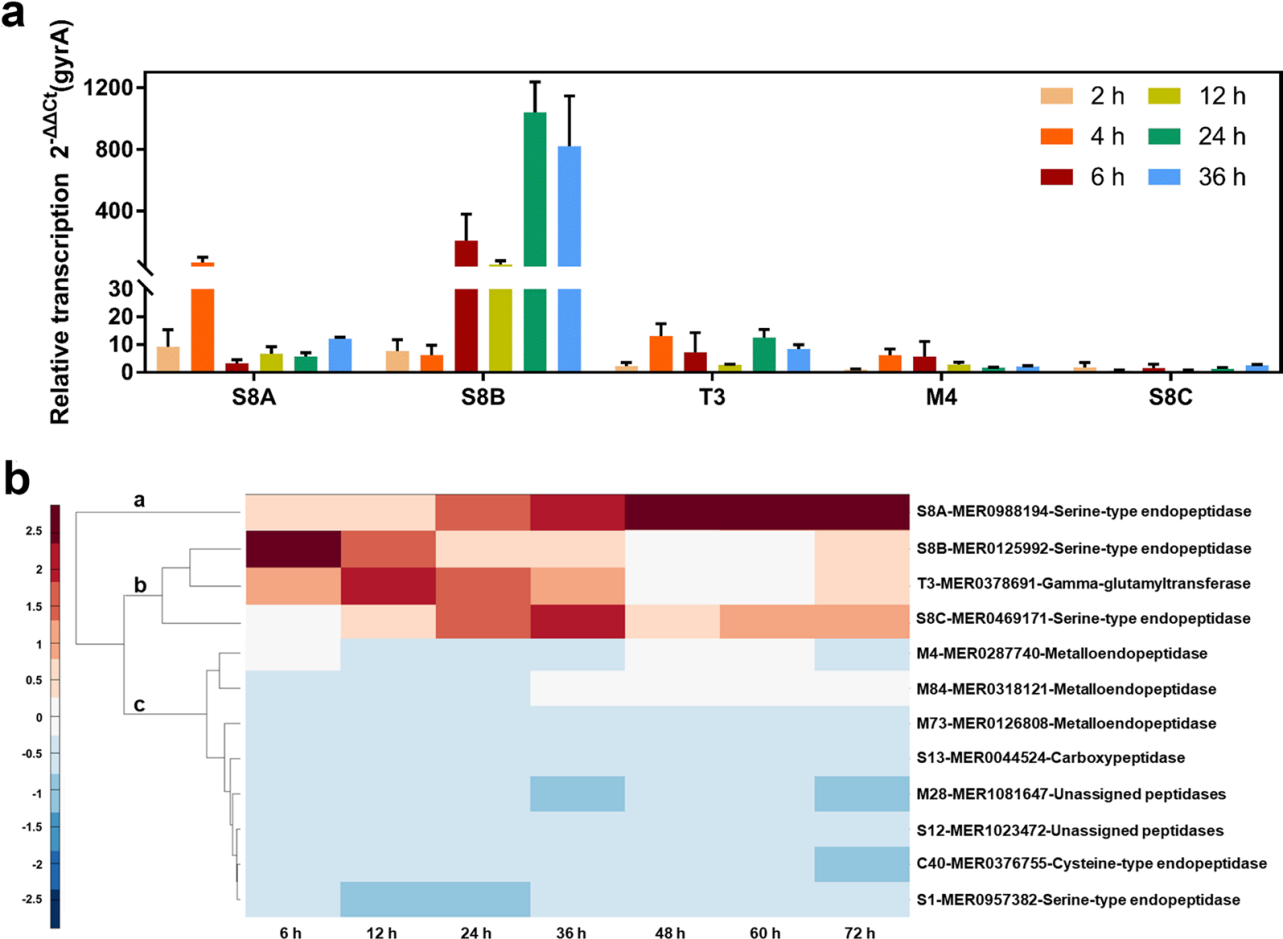
Transcriptome and proteome analysis of keratin-degrading enzyme components.** a** The expression levels of key proteases were detected by RT-qPCR. **b** Heat map comparison of the expression patterns of selected proteases detected in the secretomes of *Bacillus* sp. CN2 during growth on feather keratin at 6, 12, 24, 36, 48, 60, and 72 h

In general, microorganisms secrete a large number of extracellular proteases during growth, especially when nutrition is limited, to obtain nitrogen sources for their growth and metabolism. To confirm that proteases play an important role in feather degradation, several genes were selected to validate the transcriptional expression levels by real-time quantitative PCR (RT-qPCR). The expression profiles of S8A, S8B, and S8C serine endopeptidases, T3 γ-glutamyltransferase, and M4 metalloproteinase were detected (Fig. 6a). S8A and S8B were preferentially transcribed in the early stage of fermentation (2 h), S8A transcription reached the highest level at 4 h, and then began to decrease, while S8B maintained a high level until 36 h of fermentation. By contrast, S8C remained at a low expression level during the whole fermentation process. Furthermore, during the 0–36 h fermentation period, T3 γ-glutamyltransferase maintained a certain level of transcriptional expression. The potential role of bacterial γ-glutamyltransferase in keratinolysis has been suggested for *B. subtilis* CH-1 [4]. This enzyme cleaves the bonds between the ε-amine of lysine and the γ-glutamyl of glutamine, an isopeptide bond found in many keratinous materials [11]. The expression level of M4 metalloendopeptidase increased gradually in the first 6 h and then decreased. That is, during the first 6 h of fermentation, S8B, T3, and M4 were mainly involved and may, therefore, be important in the initial degradation of keratin.

Proteome analysis can accurately analyze changes in extracellular enzyme species. A total of 12 secretory proteases (including five serine endopeptidases, two metal endopeptidases, 1 cysteine endopeptidase, one exopeptidase, one Omega peptidase and two unclassified peptidases) and 22 intracellular proteases were detected in the secretome by LC–MS/MS (Fig. 6b and Additional file 2: Table S2). The expression levels of endopeptidases were much higher than that of exopeptidase throughout the fermentation. Most endopeptidases are neutral or alkaline serine proteases, and the relative expression of an acid cysteine endopeptidase is small (< 0.15%), which may correlate with the growth of this bacterium in a moderately alkaline saprophytic habitat. These phenomena were consistent with the results of a previous study [30].

Proteases are classified into three categories based on their secretion levels: Category a, protease secretion is continuously elevated; Category b, the concentration of protease is decreased or first increased and then decreased; Category c, proteases secretion is maintained at a low level. S8B was secreted in large quantities during the initial stage of fermentation, then decreased from 37.00% at 6 h to 14.95% at 24 h. Interestingly, T3 γ-glutamyltransferase was secreted at a relatively high level at 12 h and then decreased after 36 h. γ -glutamyltransferase catalyzed the transfer of the γ-glutamyl on glutathione to different α amino acids to generate free cysteine groups, which act as strong reducing agents to participate in the thiolysis of keratin, ultimately weakening the mechanical stability of keratin and, hence, yielding more efficient keratinolytic attack [11, 40]. It is speculated that S8B may be a keratinase that is first secreted under the induction of soluble proteins in the fermentation broth, destroys the dense structure of feather keratin, and exposes more protease contact sites. S8C also began to secrete and participate in the degradation of keratin after 24 h. Along with the degradation of keratin, soluble peptides in the fermentation broth increased, and as inducers, stimulated the secretion of S8A, and further degraded keratin into small molecule polypeptides. In addition, a small amount of M4 metalloendopeptidase, which preferentially degrades hydrophobic amino acids, was also involved in the destruction of the hydrophobic region of feather keratin in the early stage of fermentation.

When cultured for 36 h, 24 intracellular proteases were detected in strain CN2, which may reflect the release of cellular contents into the culture medium following bacterial cell death. LC–MS/MS results showed that the content of intracellular proteases was lower than that of extracellular proteases, but the variety of enzyme types was greater (Additional file 2: Table S2). These aminopeptidases with relatively high expression levels were mainly distributed among the M42, M20, M29, M17, and M55 families, and were sequence-specific enzymes (Additional file 1: Fig. S2). The specific peptides that are transported to cells can be accurately identified and further degraded into free amino acids, so that the strains can flexibly adjust the balance of hydrolytic activity of oligopeptides and amino acids according to their own nutritional needs.

### Product analysis of the feather keratin degradation system

**Fig. 7:**
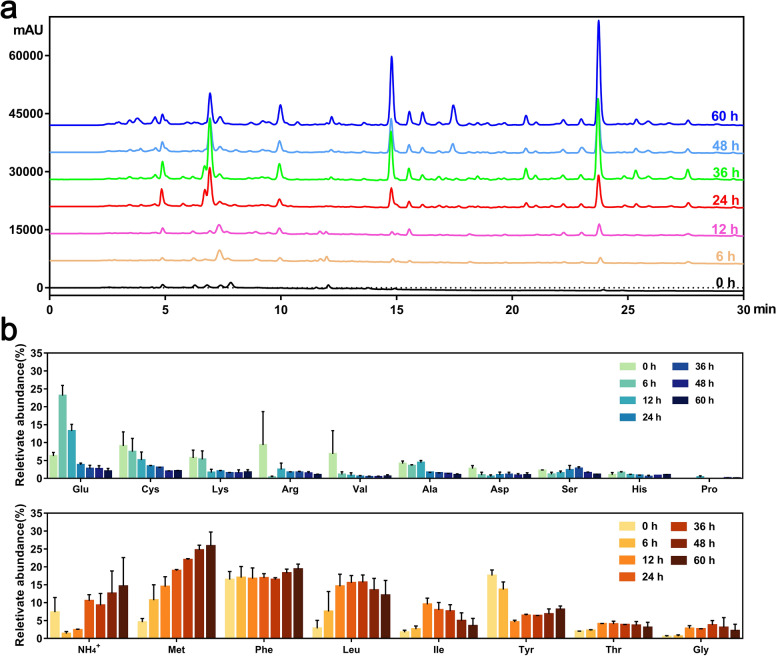
Changes in the degradation products of feathers during enzymatic hydrolysis. **a** Analysis of soluble peptides in feather hydrolysates by HPLC. **b** Analysis of free amino acids in feather hydrolysates. 0 h indicates no inoculation of bacteria. The sampling times were 6, 12, 24, 36, 48, and 60 h. To aid interpretation, HPLC data are displayed as average values

The feather hydrolysates of strain CN2 were analyzed by HPLC. Virtually no soluble peptides were detected in the sterilized culture medium. Peptides started to be detected in the fermentation broth after 6 h of proteolysis and increased with time. When most of the feathers (86.70%) were degraded at 24 h, the content and types of peptides in the hydrolysates increased rapidly. Subsequently, the concentration of polypeptides increased continuously under the action of keratinase and other proteases secreted by strain CN2. After 72 h of fermentation, 10 significant peptide peaks were detected in the degradation system (Fig. 7a). It is possible that the low secretion of M4 metalloendopeptidase, which preferentially degrades hydrophobic amino acids, leads to the generation of residues of these polypeptides. The molecular weight of keratin is usually reported to be between 3 and 10.4 kDa [41]. After hydrolysis, the molecular weight of soluble peptides was in the range of 166 to 1043 Da (Additional file 1: Fig. S3), indicating that the proteases secreted by strain CN2 effectively degraded feather keratin.

In addition, the types and contents of free amino acids in each fermentation period were tested. Within 24 h of rapid feather degradation, feather keratin was gradually hydrolyzed and a low concentration of free amino acids was produced under the synergistic action of S8B serine endopeptidases and T3 γ-glutamyltransferase. After 36 h, M4 metalloendopeptidase bound to the exposed keratin and performed further hydrolysis to produce hydrophobic amino acids, such as methionine, phenylalanine, leucine, isoleucine, and tyrosine (Fig. 7b), which was consistent with the fact that feathers contain approximately 50%–60% hydrophobic amino acids [42]. However, the contents of certain amino acids (Lysine, Tyrosine, Glutamic acid, Cysteine, and Alanine) in the fermentation broth were significantly lower than those in the non-fermentation broth. It is speculated that the content of these amino acids in feathers was low and/or widely absorbed and utilized by strain CN2 to meet its own growth requirements.

Simultaneously, NH_4_^+^ was also detected in the fermentation broth, and its content gradually increased with fermentation time. High concentrations of cysteine are toxic to cells, and microorganisms capable of degrading keratin must be able to balance cysteine levels [17, 43]. Consistent with this, only a small amount of cysteine was detected in the feather fermentation broth. At 60 h of fermentation, the total amount of amino acids began to decrease, at which time the bacteria entered the decay stage, so enzymatic hydrolysis was gradually weakened, and the growth of residual bacteria continued to consume amino acids in the fermentation broth. Finally, after the fermentation process, the proportion of essential amino acids increased, thus realizing the potential of keratin-rich waste to be converted into high-value biological products [17]. This indicated that *Bacillus* sp. CN2 is an excellent keratin-degrading bacterium and its fermentation products can be used as animal feed additives and biological fertilizers.

## Discussion

The production of large-scale industrial and agricultural waste is an increasing environmental burden. The use of renewable biotechnology to convert such waste into high-value resources is, therefore, urgently needed. Previous studies have shown that *Bacillus* sp. CN2 isolated from the compost of chicken manure has significant protein degradation capacity [30]. The biochemical results of this study showed that the extracellular protease produced by strain CN2 had great potential for hydrolyzing keratin because it degraded 86.7% of intact feather keratin within 24 h and was able to use these hydrolysates as nutrients (Figs. 1 and 2). A similar phenomenon has been observed for other keratinolytic microbes distributed in different ecological niches [20, 34]. *Bacillus* is a major producer of keratinase, which may reflect its excellent adaptability to highly dense keratin [44].

To take advantage of the high nitrogen content of keratinous waste, keratinase can be used to convert such waste into high-value biological products [19]. The keratinase activity of strain CN2 is reportedly significantly higher than for most other microorganisms analyzed, such as *B. licheniformis* ALW1 [31], *B. cereus* B5esz [32], and *B. subtilis* MBF11 [33], for which the maximum keratinase activity levels were 72.2, 10.0, and 78.0 U/mL, respectively. The environmental conditions and fermentation medium may have contributed to the significant disparity in keratinase activity among several bacteria [45], or some microorganisms may be under nutritional stress and require faster degradation substrates as nutrients [46]. Differential expression of the transcriptome (Fig. 6a) may reflect the fact that the three serine endopeptidases are regulated by different transcription factors or that one protease is hydrolyzed by other proteases as a result of a lack of stability at the protein level. Keratinases are mostly serine or metalloproteases that show a broad spectrum of activity and exhibit specificity for hydrophobic and aromatic residues at the P1 site [47]. Mass spectrometry analysis in this study showed that strain CN2 could express and secrete a variety of extracellular proteases in the feather medium, mainly comprising serine and metal endopeptidases (Fig. 6b). Interestingly, strain CN2 secreted higher concentrations of T3 γ-glutamyltransferase when growing on feather keratin relative to other natural substrates such as wheat bran, corn bran, corn steep liquor, and maize protein powder [30]. *B. subtilis* γ-glutamyltransferase has reported efficacy in cleaving the γ-glutamyl bond of glutathione to release cysteinylglycine as a sulfur source [40]. These enzymes can bind to hydrophobic substrates and affect disulfide bonds. Under the action of these proteases, casein, gelatin, soybean cake meal, and other types of protein substrates are rapidly degraded (Fig. 3c), indicating that this strain has high protein degradation potential. These characteristics highlight the potential of this bacterium in the recovery of keratinous waste biomass and industrial production.

**Fig. 8:**
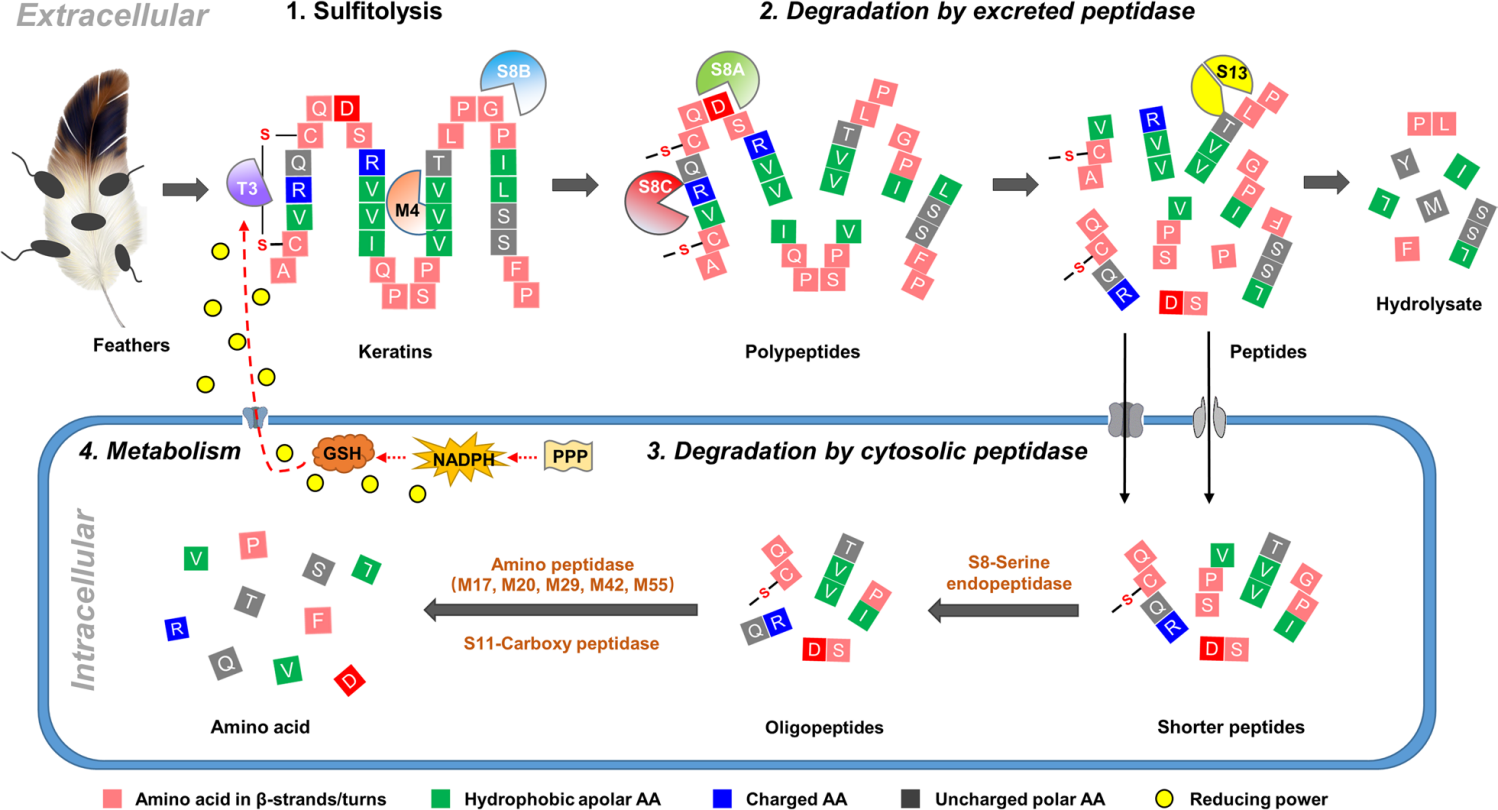
Schematic representation of the keratin degradation mechanism of *Bacillus* sp. CN2

The disulfide bonds present in keratin are a key factor hindering it degradation. Therefore, most keratinases can only degrade keratin after breaking the disulfide bonds (reduction reaction) [17]. Consequently, keratin hydrolysis may include two steps: sulfitolysis and proteolysis [17, 24]. In vitro experiments showed that keratin could not be degraded effectively by relying on the presence of keratinase from CN2 with Na_2_SO_3_ as a reducing agent, whereas DTT, improved the degradation process of keratin (Fig. 4). It also suggested that live cell adhesion was important for the full degradation of keratin, because the continuous supply of reducing agents from living cells could help to break the disulfide bridge, which was also reported in a previous study [48]. Although the CN2 genome encodes six disulfide reductases, no relevant reductases were detected in the proteome (Additional file 1: Table S1 and S2). The abundantly expressed T3 γ-glutamyltransferase likely provides the reducing force to break keratin disulfide bonds and may be the only reducing force for this degradation system. Once T3 γ-glutamyltransferase is involved, the feather keratin is rapidly degraded in a short time (Fig. 8). This result indicates that only the combination of T3 γ-glutamyltransferase and endopeptidase can effectively degrade keratin, which is different from the keratin degradation mode of other *Bacillus*, which use disulfide reductase to utilize keratin [37].

The treatment of waste keratin is an issue of concern [38]. It was reported that purified keratinase was unable to effectively degrade keratin because of lack of necessary synergistic factors, such as disulfide reductase [17]. By contrast, keratinase-producing microorganisms are favored because of their requirement for simple culture conditions, low cost, and high capacity for enzyme production [20, 48]. With improvements in the keratinolytic microbial genome and proteome database, the mechanism of keratin hydrolysis by keratinase-producing microorganisms based on degradomics will become clearer in the future.

## Conclusions

By integrated functional-degradomics technology, this study revealed that *Bacillus* sp. CN2 had a strong ability to degrade feather keratin, and the hydrolysates contained abundant soluble peptides and essential amino acids. This bacterium exhibits broad substrate specificity, and can completely degrade soluble and insoluble substrates within 2 min. A highly efficient keratin degradation system was discovered by extracellular proteomics analysis that predominantly included one T3 γ-glutamyltransferase, three S8 serine endopeptidases, and one M4 metalloendopeptidase. These synergistic enzymes appeared to denature and then hydrolyze keratin. Therefore, a systematic study of keratin degradation mediated by the keratinase-producing microorganism will lay a theoretical foundation for the efficient industrialized transformation of natural keratinous waste into high-value products.

## Materials and methods

### Feather preparation and calculation of the degradation rate

Chicken feather waste was collected from Yu cheng (Dezhou, China) poultry farms. It was first washed with tap water several times until all skin and dust had been removed, then with anhydrous ethanol twice, followed by soaking overnight, and finally with deionized water three times to remove anhydrous ethanol, followed by drying in an oven for 24 h at 65 °C. The dried feathers were then stored in airtight bags for subsequent analysis.

The feather degradation rate was defined as the change in feather dry weight before and after degradation. The fermentation broth was passed through the filter paper to remove the non-degraded feather residue, was washed with deionized water 2 − 3 times to completely remove soluble substances and attached bacteria, and then dried in an oven at 65 °C to a constant weight. The following formula was used to calculate the feather degradation rate: feather degradation rate (%) = 100 × (B − A) / B, where B is the dry weight of the feathers before degradation and A is the dry weight of the feathers after degradation.

### Shake-flask fermentation of feather keratin and the preparation of cell-free fermentation broth

*Bacillus* sp. CN2 was grown in feather fermentation medium (1% *w/v* feather, 0.5% *w/v* sucrose, 0.5% *w/v* trisodium citrate dihydrate, 0.5% *w/v* K_2_HPO_4_, 0.01% *w/v* MgSO_4_·7H_2_O, 0.01% *w/v* CaCl_2_·2H_2_O, pH 7.0) at 37 °C with shaking at 200 rpm.

The fermentation broth was collected at the specified time interval. The suspension was filtered through eight layers of gauze to remove the feather powder floating on the surface, and was then centrifuged for 5 min at 10,000 × g. The obtained crude enzyme was used for subsequent experimental analysis.

### Determination of the soluble protein content

The soluble protein content was determined by the Coomassie brilliant blue G-250 method with bovine serum albumin (BSA) as the standard protein [49].

### Assay of keratinase activity

Evaluation of isolates for keratinase activity was conducted according to a previously described method by Jaouadi and colleagues with slight modification [50]. The crude enzyme (0.5 mL), diluted with 50 mM Tris–HCl, was added to 0.5 mL of 10 g/L soluble keratin substrate, and shock incubated at 40 °C for 1 h. Trichloroacetic acid (TCA, 2 mL, 0.4 mol/L) was added to terminate the reaction, followed by centrifugation for 10 min at 10,000 ×*g* to remove the residual substrate. The supernatant was filtered through a 0.22 μm membrane and the absorbance at 280 nm was measured using a spectrophotometer. The controls were prepared by adding the TCA prior to the enzyme. One unit (U/mL) of keratinolytic activity was defined as the amount of enzyme that caused an increase of 0.01 OD at 280 nm per min under the assay conditions calculated by the equation below. Three sets of parallel replicates were conducted in all experiments.

### Determination of the sulfate concentration

The sulfate content was determined by barium chromate spectrophotometry. Briefly, 2 mL of fermentation broth supernatant was mixed with 8 mL of deionized water and 5 mL of barium chromate suspension. After standing for 30 min, the suspension was mixed with 1.0 mL of calcium ammonia solution and 10 mL of 95% ethanol. After shaking vigorously for 1 min, the suspension was filtered through medium speed quantitative filter paper, and the absorbance at 420 nm was measured. Blank fermentation broth was included as a control to calculate the SO_4_^2−^ content in the culture broth.

### Determination of the sulfite concentration

The concentration of sulfite was determined by the hydrochloric acid pararosaniline method. Briefly, 1 mL of fermentation broth supernatant was evenly mixed with 2 mL of formaldehyde pararosaniline solution, and the absorbance at 550 nm was measured. Blank fermentation broth was included as a control, and the content of SO_3_^2−^ in the fermentation broth was calculated.

### Determination of the thiol concentration

The thiol concentration was determined by the method reported by Ellman and colleagues [25]. Briefly, 50 μL of 5,5ʹ-dithiobis (2-nitrobenzoic acid) (DTNB, 4 mg/mL; Sigma–Aldrich, St. Louis, MO, USA) in 100 mM phosphate buffer, pH 8.0, was mixed with 500 μL of distilled water. Then, 200 μL of fermented broth was added to the mixture, and allowed to stand for 10 min at room temperature. The yellow-colored 2-nitro-5-thiobenzoic acid (TNB) that formed upon reduction of DTNB was measured at an absorbance of 412 nm. Non-inoculated media served as a control. Every experiment was repeated in triplicate and the results are shown as the mean ± standard deviation.

### Analysis of soluble peptides and amino acids in the fermentation broth

The clarified, degraded feather suspension was obtained by filtration with eight layers of gauze and centrifugation for 10 min at 10,000 × g and 4 °C. The supernatant was mixed with an equal volume of 1% TFA, and the mixture was incubated for 30 min at 4 °C and centrifuged for 15 min at 13,000 × g. The supernatant was retained and filtered through a 0.22 μm membrane filter. Free amino acid components in the feather hydrolysate supernatants sampled at different time points were measured using an amino acid analyzer (Model L-8900, Hitachi, Tokyo, Japan).

Peptides were detected using the HPLC system (Shimadzu, Kyoto, Japan) equipped with a UV detector and a Shim-pack GIST C18 column (250 mm × 4.6 nm i.d., 5 μm particle size; Shimadzu, Kyoto, Japan). The mobile phase A was 0.1% TFA and mobile phase B was acetonitrile mixed with 0.1% TFA. The flow rate was 1.0 mL/min. The detection wavelength was set at 240 nm at ambient temperature.

### Feather structural studies by scanning electron microscopy (SEM)

The biodegradable feathers were observed by SEM following the procedures described by Gupta and Singh [51]. Briefly, the residual feathers at different fermentation points were collected, washed with distilled water and dried for 24 h at 60 °C. After the sample was fully dried, the cover glass was adhered to the sample table fixed with conductive tape, plated with gold for 5 min, and observed under a field emission scanning electron microscope (Quanta 250 FEG).

### Transcriptomic and proteomic analysis of extracellular functional proteases

The fermentation liquid was sampled (1 mL) at different time points, and total RNA was obtained using the bacterial total RNA extraction kit (Tiangen, Beijing). The quality and quantity of the RNA were measured using the NanoPhotometer-N60 (Implen, German) and electrophoresis on a 1% agarose gel. Using 1 μg of RNA as a template, the total cDNA was obtained by reverse transcription with the FastKing RT kit (with gDNase) (Tiangen), and stored at − 80 °C prior to use. qRT-PCR was performed using SYBR qPCR Master Mix (Vazyme) on LightCycler 480 instrument (Roche, Basel, Switzerland). The gyrase A (*gyrA*) gene was employed as a housekeeper gene and the corresponding primers, gyrA-F (5'-CATTCTGGATATGCGCCTTCAG-3') and gyrA-R (5'-GTCACGATTTCAGTGCGTCTC-3') were used to amplify the gene.

Analysis of proteins by liquid chromatography tandem mass spectrometry (LC–MS/MS), which was consistent with the literature report [30].

## Supplementary Information

**Additional file 1: Figure S1.** Changes in the content of cysteine in the reaction system were detected by electrochemical detection. **Figure S2.** Substrate sequence spectra of different peptidase families. The binding sites of peptide substrates that interact with protease binding pockets S4-S4’ were shown as P4, P3, P2, P1, P1’, P2’, P3’, P4’ from N-terminal (N) to C-terminal (C) along the x-axis. The observed frequency for each amino acid in each position was calculated as a bit score and shown on the y-axis. Substrate cleavage occurred between P1 and P1’. Only those with > 10 cleavages in the MEROPS collection are shown as substrate sequence profiles of different peptidase families. **Figure. S3** MALDI-TOF MS analysis of enzymatic hydrolysates of feathers keratin by *Bacillus* sp. CN2.

**Additional file 2: Table S1.** Disulfide reductases and their different reaction types in the *Bacillus* sp. CN2 genome. **Table S2.** The expression dynamics of functional enzyme components secreted by *Bacillus* sp. CN2 growth on chicken feather.

## Data Availability

The datasets supporting the conclusions of this article are included within the article and its additional files.

## Abbreviations

TCA: Trichloroacetic acid
DTNB: 5,5ʹ-Dithiobis (2-nitrobenzoic acid)
TFA: Trifluoroacetic acid
HPLC: High-performance liquid chromatography
SEM: Scanning electron microscopy
RT-qPCR: Quantitative real-time PCR
LC–MS/MS: Liquid chromatography–tandem mass spectroscopy
