# Supplementary material for: Insights into the keratin efficient degradation mechanism mediated by Bacillus sp. CN2 based on integrating functional degradomics
Source: Biotechnol Biofuels Bioprod. 2023 Apr 4;16:59. doi: 10.1186/s13068-023-02308-0 (PMC10071666; doi:10.1186/s13068-023-02308-0)
Supplement: Supplementary file 1 — Additional file 1: Figure S1. Changes in the content of cysteine in the reaction system were detected by electrochemical detection. Figure S2. Substrate sequence spectra of different peptidase families. The binding sites of peptide substrates that interact with protease binding pockets S4-S4’ were shown as P4, P3, P2, P1, P1’, P2’, P3’, P4’ from N-terminal (N) to C-terminal (C) along the x-axis. The observed frequency for each amino acid in each position was calculated as a bit score and shown on the y-axis. Substrate cleavage occurred between P1 and P1’. Only those with > 10 cleavages in the MEROPS collection are shown as substrate sequence profiles of different peptidase families. Figure. S3 MALDI-TOF MS analysis of enzymatic hydrolysates of feathers keratin by Bacillus sp. CN2. [file 13068_2023_2308_MOESM1_ESM.docx]

**Supplementary information**

**Insights into the keratin efficient degradation mechanism mediated by *Bacillus* sp. CN2 based on integrating functional degradomics**

Yuhong Lai^1^, Xiuyun Wu^1^, Xianliang zheng^2^, Weiguang Li^1^, Lushan Wang^1^*

^1^State Key Laboratory of Microbial Technology, Institute of Microbial Technology, Shandong University, Qingdao, Shandong, 266237, China

^2^Angel Yeast Inc, Yichang, Hubei, 443003, China

***Corresponding author:** Professor Lushan Wang

**E-mail address:** lswang@sdu.edu.cn

**Tel:** +86-532-58631570

**Full postal address:** State Key Laboratory of Microbial Technology, Shandong University, 72 Binhai Road, Qingdao, 266237, China

Yuhong Lai: [Laiyh0921@163.com](mailto:Laiyh0921@163.com)

Xiuyun Wu**:** [wuxiuyun3353@163.com](mailto:wuxiuyun3353@163.com)

Xianliang zheng: zhengxl@angelyeast.com

Weiguang Li: sdsyli@163.com


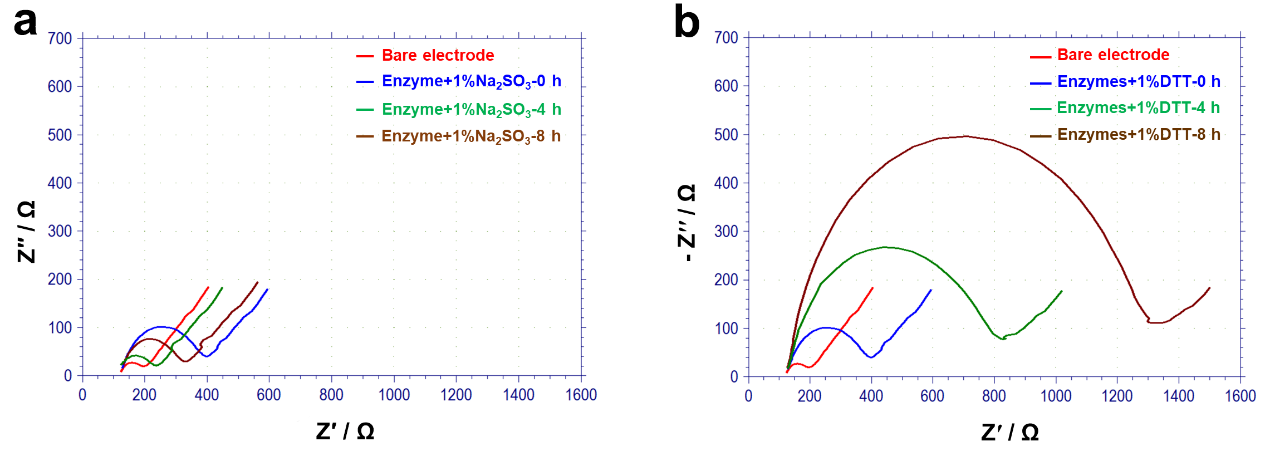


**Fig. S1** **Changes in the content of cysteine in the reaction system was detected by electrochemical detection.**


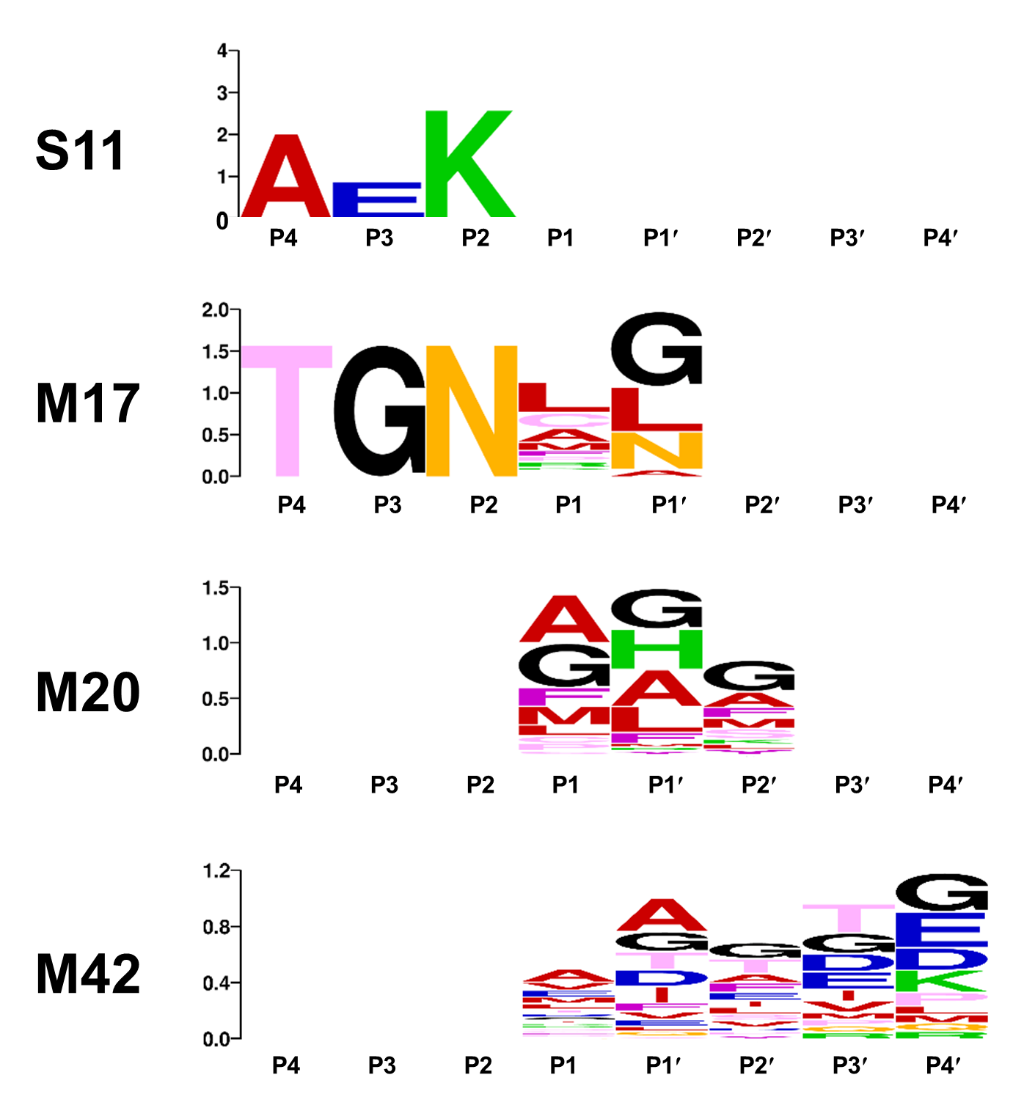


**Fig. S2** **Substrate sequence spectra of different peptidase families**. The binding sites of peptide substrates that interact with protease binding pockets S4-S4’ were shown as P4, P3, P2, P1, P1’, P2’, P3’, P4’ from N-terminal (N) to C-terminal (C) along the x-axis. The observed frequency for each amino acid in each position was calculated as a bit score and shown on the y-axis. Substrate cleavage occurred between P1 and P1’. Only those with > 10 cleavages in the MEROPS collection are shown as substrate sequence profiles of different peptidase families.


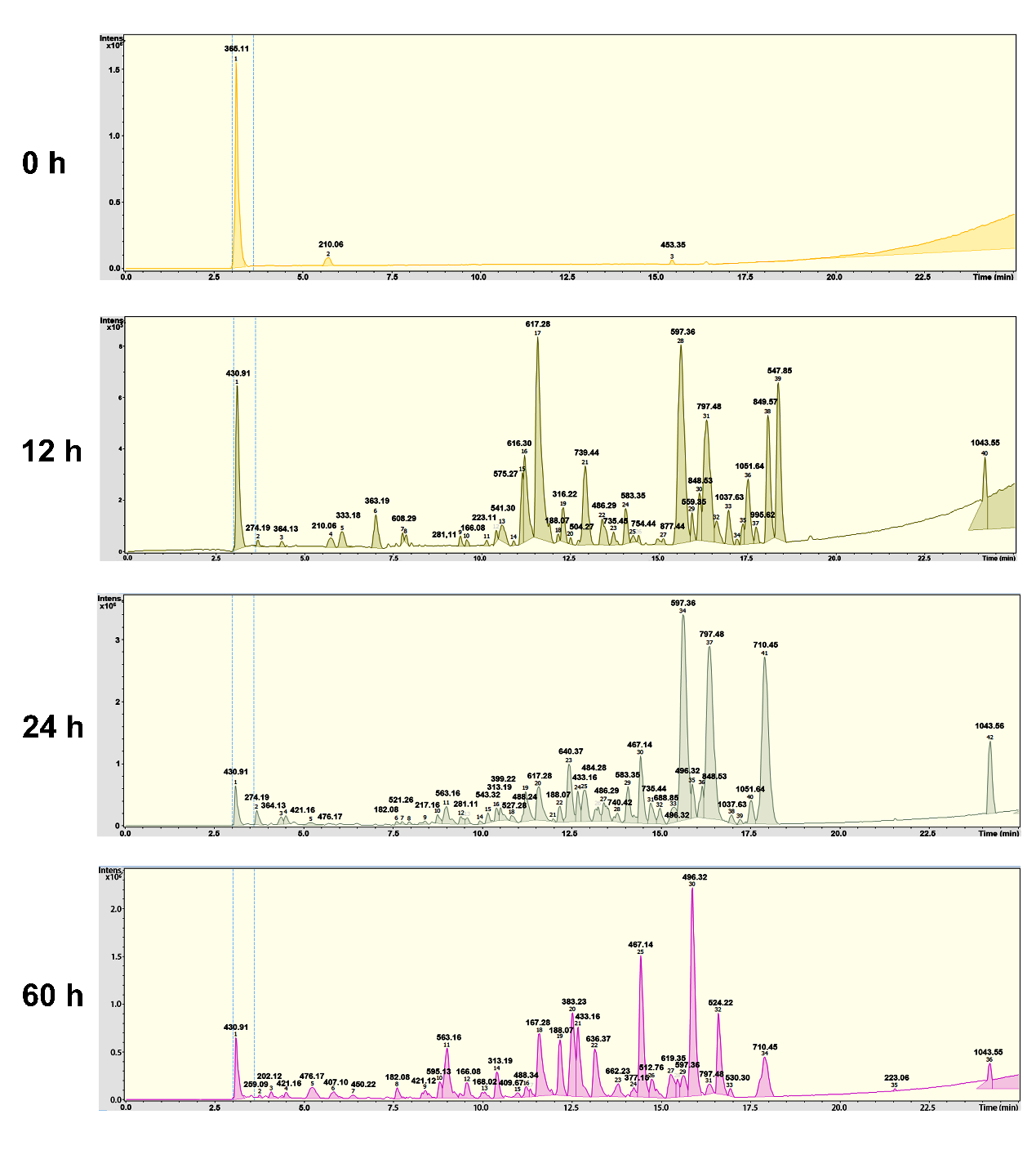


**Fig. S3 MALDI-TOF MS analysis of enzymatic hydrolysates of feathers keratin by *Bacillus* sp. CN2.**
